# Dark-rim-free ungated first-pass perfusion CMR with 3-Slice end-systolic imaging: initial experience

**DOI:** 10.1186/1532-429X-16-S1-P177

**Published:** 2014-01-16

**Authors:** Behzad Sharif, Reza Arsanjani, Aryeh Shalev, Rohan Dharmakumar, C Noel Bairey Merz, Daniel S Berman, Debiao Li

**Affiliations:** 1 Biomedical Imaging Research Institute, Cedars-Sinai Medical Center, Los Angeles, California, USA; 2 Heart Institute, Cedars-Sinai Medical Center, Los Angeles, California, USA

## Background

First-pass perfusion (FPP) cardiac MR (CMR) imaging has been shown to have a high performance for diagnosis of coronary artery disease (CAD). Reliability of FPP imaging, however, is hindered by dark-rim artifacts (DRAs) and the need for near-perfect ECG gating. The latter can be challenging in the presence of arrhythmias or heart-rate variations during stress. Moreover, end-systolic (ES) imaging has recently been shown to provide improved visualization of subendocardial defects [1]. We developed an innovative ungated FPP technique capable of simultaneously eliminating DRAs [2,3] and enabling reconstruction of all slices at ES. We hypothesized that the developed method achieves DRA-free imaging and high accuracy in patients with suspected CAD, using nuclear myocardial perfusion imaging (MPI) as the reference.

## Methods

**Figure 1:**
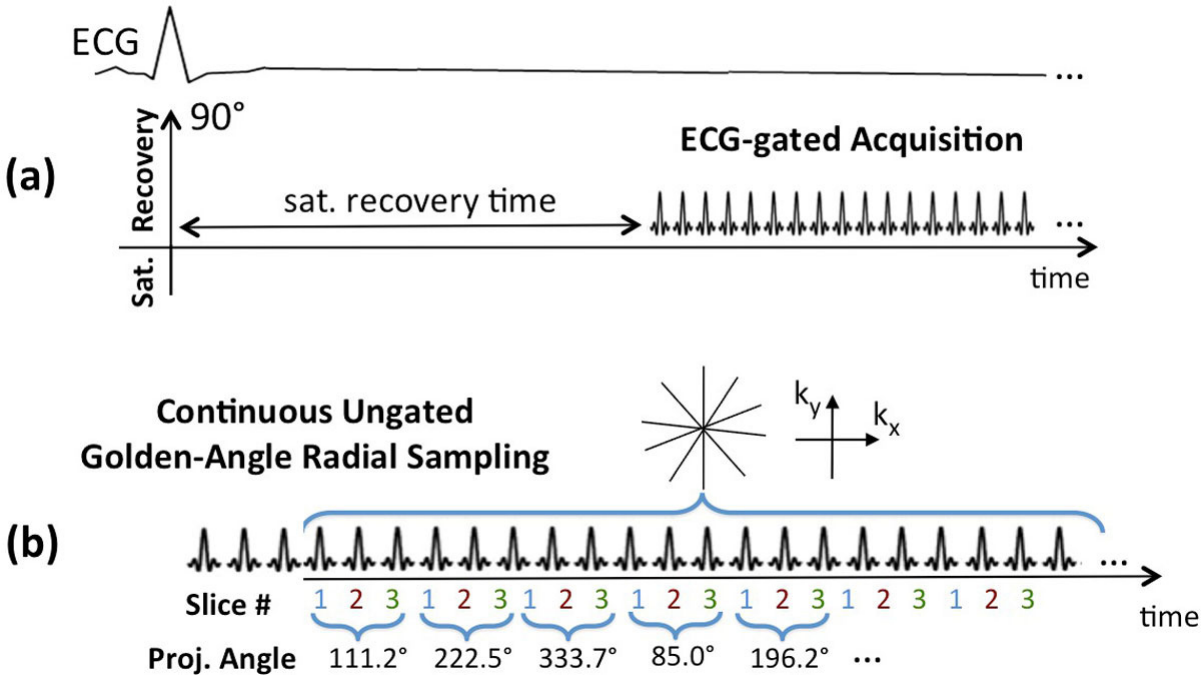
**(a) Schematic for a conventional first-pass perfusion (FPP) pulse sequence with saturation recovery (SR) preparation and ECG synchorinization**. (b) Schematic for the proposed Ungated Cine FPP pulse sequence using non-ECG-gated continuous golden-angle radial acquisition that is interleaved between 3 short-axis slices.

Based on the so-called "Ungated Cine FPP" approach [4], a multi-slice magnetization-driven [5-7] method was developed for ungated FPP imaging. Figure 1 highlights the main differences of the proposed pulse sequence compared with the conventional FPP method. All scans were performed on a 3T clinical scanner using an ungated RF-spoiled GRE sequence with continuous golden-angle radial acquisition as in Figure 1b (flip angle = 21°, resolution: 1.7 × 1.7 × 10 mm). The reconstruction method used automatic self-gating and optimally apodized [3] compressed sensing for DRA-free accelerated reconstruction. Normal subjects (n = 6) were studied using both the proposed and conventional methods. Patients (n = 9) with suspected CAD on the basis of recent abnormal SPECT/PET MPI underwent adenosine stress/rest FFP CMR. Three patients returned for a second study using the conventional method.

## Results

**Figure 2:**
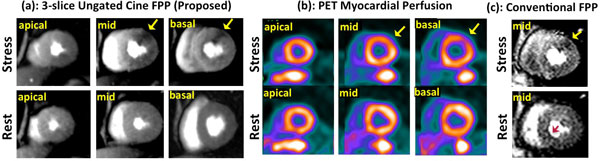
**Representative images from the CAD patient study (top row: vasodilator stress; bottom row: rest, yellow arrows point to perfusion defects)**. (a) End-systolic 3-slice images for the proposed Ungateed Cine FPP method (1.7 × 1.7 mm^2 ^in-plane resolution). (b) Corresponding PET myocardial perfusion slices (mild ischemia qith 6% stress-induced defect in the anter-lateral wall at mild-basal slices). (c) Conventional ECG-gateed SR-prepared FPP images (red arrow points to dark-rim artifact). The Ungated Cine FPP images are in strong agreement with the PET study and have higher quality than conventional FPP.

The ungated FPP studies in normal subjects were all of high quality and demonstrated normal perfusion. A representative patient study is shown in Figure 2. Stress-induced hypoperfusion was observed in the ES Ungated Cine FPP images, corresponding to a reversible defect on PET MPI (Figure 2b). Figure 2c shows the mid slice for the conventional FPP scan. Based on nuclear MPI studies, sensitivity and specificity of the developed method were 93% and 95%, respectively. The minor disagreements can be explained by the presence of subendocardial defects and possible artifacts on SPECT. All images were reviewed and no DRAs was detected on the Ungated Cine FPP images (2 readers, consensus). However, for the 3 studies using the conventional method, mild-moderate DRA was observed in 21% of the segments.

## Conclusions

Conventional FPP methods are prone to DRAs, require accurate ECG gating, and do not provide the freedom to image all slices at ES. The developed method overcomes these challenges and is an attractive alternative with the advantage of simplicity (no gating), higher accuracy in the subendocardium (no DRAs, ES imaging), and thereby potentially improved reliability. Preliminary results in healthy volunteers and patients with suspected CAD were of high quality and showed high accuracy compared to nuclear MPI.

## Funding

Grant sponsors: National Institutes of Health grants nos. NHLBI R01HL38698 and R01HL091989. American Heart Association Postdoctoral Fellowship Award 11POST7390063.
